# Application of Sewage Sludge in a Rice (*Oryza sativa* L.)-Wheat (*Triticum aestivum* L.) System Influences the Growth, Yield, Quality and Heavy Metals Accumulation of Rice and Wheat in the Northern Gangetic Alluvial Plain

**DOI:** 10.3390/life12040484

**Published:** 2022-03-27

**Authors:** Surendra Singh Jatav, Satish Kumar Singh, Manoj Parihar, Amnah Mohammed Alsuhaibani, Ahmed Gaber, Akbar Hossain

**Affiliations:** 1 Department of Soil Science and Agricultural Chemistry, Institute of Agricultural Sciences, Banaras Hindu University, Varanasi 221005, Uttar Pradesh, India; surendra.jatav1@bhu.ac.in; 2 ICAR-Vivekananda Parvatiya Krishi Anusandhan Sansthan, Almora 263601, Uttarakhand, India; manojbhu7@gmail.com; 3 Department of Physical Sport Science, College of Education, Princess Nourah bint Abdulrahman University, P.O. Box 84428, Riyadh 11671, Saudi Arabia; amalsuhaibani@pnu.edu.sa; 4 Department of Biology, College of Science, Taif University, P.O. Box 11099, Taif 21944, Saudi Arabia; a.gaber@tu.edu.sa; 5 Department of Agronomy, Bangladesh Wheat and Maize Research Institute, Dinajpur 5200, Bangladesh

**Keywords:** plant height, yield, protein content, rice-wheat cropping system, sewage sludge

## Abstract

For a sustainable and profitable agriculture production system, balanced and integrated use of nutrients is a key strategy. In addition, partial replacement of chemical fertilizers with organics ones reduces both environmental concerns and economic costs and provides greater soil health benefits. With this hypothesis, an experiment was conducted to assess the yield and economic benefits of a rice-wheat cropping system (RWCS) as influenced by the joint application of sewage sludge (SSL) and fertilizer. The treatments comprised: without fertilizer or SSL; 100% recommended dose of fertilizers (RDF); 100% RDF + 20 Mg ha^−1^ SSL; 100% RDF + 30 Mg ha^−1^ SSL; 50% RDF + 20 Mg ha^−1^ SSL; 60% RDF + 20 Mg ha^−1^ SSL; 70% RDF + 20 Mg ha^−1^ SSL; 50% RDF + 30 Mg ha^−1^ SSL; 60% RDF + 30 Mg ha^−1^ SSL and 70% RDF + 30 Mg ha^−1^ SSL. The experiment was laid out in a randomized block design with three replications. The result of our study indicate that the highest percent increase in mean plant height i.e., ~14.85 and ~13.90, and grain yield i.e., ~8.10 and ~18.90 for rice and wheat, respectively, were recorded under 100% RDF + 30 Mg SSL ha^−1^ treatment compared to 100% RDF, while 70% RDF + 20 Mg ha^−1^ SSL produced a statistically equivalent grain yield of 100% RDF in RWCS. The application of 20 and 30 Mg SSL ha^−1^ along with recommended or reduced fertilizer dose, significantly increased the heavy metal content in plant and soil systems above that of 100% RDF, but this enhancement was found within permissible limits. Moreover, the reduced use of SSL i.e., 20 Mg SSL ha^−1^, resulted in lower heavy metal content in grain and soil than did the 30 Mg ha^−1^ SSL treatment, but significantly higher than in the absolute control or 100% RDF treatment. In summary, the use of 20 Mg ha^−1^ SSL along with 70% RDF provided a safer, profitable and sustainable option in a rice-wheat cropping system in the middle Ganegatic alluvial plain.

## 1. Introduction

A rice-wheat cropping system (RWCS) is the main cropping pattern occupying 24 million hectares (Mha) of cultivated land in the Indo-Gangetic Plains (IGP) in South Asian subtropics and China [1]. This covers an area of 13.5 Mha in the IGP. Out of this 10, 2.2, 0.8 and 0.5 Mha lie in India, Pakistan, Bangladesh, and Nepal, respectively, and the remainder of 10.5 Mha is found in China [1,2,3]. Therefore, it was judged suitable to use with a test crop in the present study. In RWCS, nutrient removal occurs more than replenishment with the application of chemical fertilizer [4]. This situation is anticipated to worsen in the future, as more food needs to be produced to feed an ever-increasing population. As a result, the use of organic amendments with chemical fertilizers is to be encouraged in order to maintain soil fertility for sustainable agriculture [5]. In sustainable agricultural, the use of traditionally applied inorganic fertilizers cannot be over-emphasized because of the high fertilizer cost and their negative impact on the soil environment. Therefore, the substitution of available organic wastes is required [6,7]. The long-term and continuous uptake of nutrients from soils without adding organic manure leads to land degradation. The intensive application of inorganic fertilizers also decreases soil quality due to salt accumulation (Cl^−^ and SO_4_^2−^) in the rhizosphere zone [8]. Hence, there is an imperative need to select suitable organic manure for replacement or reduce inorganic fertilizer doses in the RWCS.

Different organic manure and waste occur in nature, but due to continuous urbanization, a vast amount of sewage sludge (SSL) is being produced which can also be used as manure for improving agricultural production and to mitigate environmental concerns [9] with economic feasibility [10]. Sewage sludge is a heterogeneous mixture of undigested organic materials such as cellulose, plant residues, oil or faecal material, and inorganic materials [11]. In developing countries, the number of sewage treatment plants is increasing due to growing urbanization and development. In India, around 100,000 million tons (Mt) SSL or soil waste is generated annually from 59 cities [12]. Sewage sludge (semi-solid material) is produced during the sewage treatment process [6]. Application of SSL in soil improves the availability of nutrients, soil water retaining capacity, soil structure, and porosity [13,14,15,16], and maintains organic matter [17,18] thereby reducing the need for synthetic fertilizer [19]. Earlier studies on SSL with chemical fertilizer application have shown improved growth, yield and yield of the crop, [20] as well as better macronutrient and micronutrient status [21] and amplification of microbial counts in the soil [7]. In agriculture, the combined use of chemical fertiliser with SSL as a source of nutrients improves soil fertility and farm profitability [22,23,24]. Integrated use of chemical fertilizers along with SSL has shown better yield performance, improvement in mineralizable nitrogen (N) and microbial biomass [12,25]. Several researchers also reported that the sludge can be used as an amendment option for degraded land, which improves soil physical properties, i.e., bulk density, micro aggregate, water retention, porosity, and hydraulic conductivity [26] as compared to inorganic fertilizer due to its carbon enriched nature. However, in addition, sludge has causes some undesirable modifications, such as a decline in pH, rise in salinity, and heavy metal content in soil [16]. Thus, its agricultural application requires monitoring to avoid the risk of heavy metal contamination in the soil and plant system [27].

The novelty of the present study is the integrated and balanced utilization of SSL and inorganic fertilizer for sustainable growth of crops. Sewage sludge has low-cost and easily available in urban areas and could substitute for farmyard manure (FYM). The current study was conducted (i) to determine the effect of joint application of SSL with chemical fertilizer on growth and yield of rice and wheat, (ii) to determine the effect of SSL with chemical fertilizer on the protein content of rice and wheat and (iii) to evaluate the effect of joint application of SSL with chemical fertilizer on bioaccumulation of heavy metals in RWCS. It is hypothesized that the joint application of SSL and fertilizer in a proper combination may positively influence the growth, yield and quality of RWCS in the middle Ganegatic alluvial plain.

## 2. Materials and Methods

### 2.1. Experimental Site

**Figure 1:**
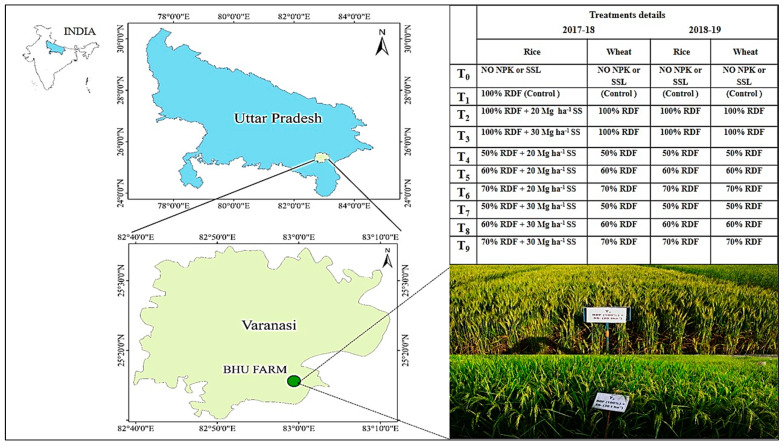
Location of the experimental site, layout and experimental view.

An experiment with two cropping cycles of rice (*Oryza sativa*; Arize 6444)–wheat (*Triticum aestivum*; HD 2967) was completed in 2015–2016 (I-rice and I-wheat) and 2016–2017 (II-rice and II-wheat). The present investigation comprised the next two cycles of rice-wheat set up in 2017–2018 (III-rice and III-Wheat) and 2018–2019 (IV-Rice and IV-wheat) without disturbing the field design of the previous experiment at the Agricultural Research Farm, Banaras Hindu University, Varanasi (UP), India. This farm is situated in the Northern Gangetic Alluvial (Inceptisol) Plain (128.93 m asl; latitude 25°19′ N, and longitude 83° E) (Figure 1).

### 2.2. Weather and Soil Condition

**Figure 2:**
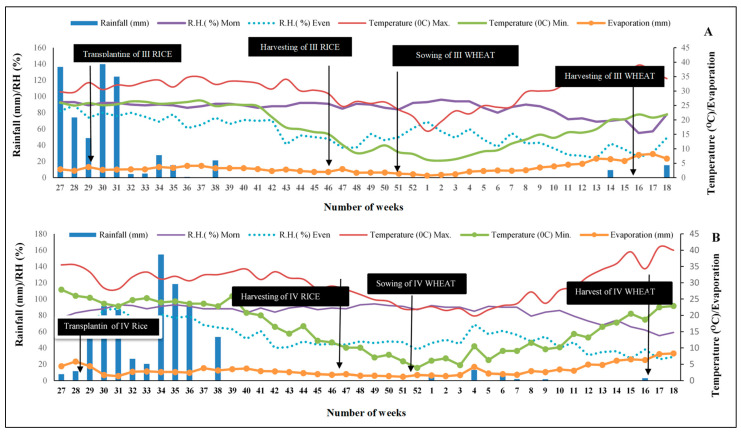
Meteorological data during the experiment in 2017–2018 (**A**) and 2018–2019 (**B**).

The region has semi-arid to sub-humid climatic conditions. Annual mean rainfall received during the experimentation was 727.75 mm and 1121.10 mm between 2017–2018 and 2018–2019, respectively, and 75% of this amount was received from June to September (Figure 2).

The experimental soil was alkaline in nature (pH 8.24), non-saline (EC 0.15 dS m^−1^), low in organic carbon content (4.60 g kg^−1^), low in available N (141.72 kg ha^−1^), medium in available P (17.42 kg ha ^−1^), medium in available K (132.74 kg ha^−1^) and medium in sulfur content (14.65 mg kg^−1^). The DTPA-extractable Fe, Cu, Zn, Mn Pb, Cd, Cr, and Ni contents in the initial soil were 42.65, 2.17, 1.02, 11.41, 0.55, 2.12, 9.24 and 6.79 mg kg^−1^, respectively.

### 2.3. Characteristics of Sewage Sludge

SSL of domestic origin was collected from a Sewage Treatment Plant (STP) in Bhagwanpur, Varanasi, in the month of May 2017. For further analysis, a composite sample was ground and passed through a 2 mm sieve and stored in a polythene bag. The SSL used in the experiment had pH 7.02, EC: 3.25 dS m^−1^, organic carbon: 7.98%, total N: 1.85%, total P: 1.40% and total K: 1.20%. According to the Council of the European Communities [28], the maximum permissible limits (MPLs) for potentially toxic elements such as Zn, Cu, Cd, Pb, Ni and Cr in sludge used in agricultural soils are 2500, 1000, 20, 750, 300 and 750 mg kg^−1^, respectively. The sludge used for the study contained 200, 247, 8, 52, 17 and 44 mg kg^−1^ of Zn, Cu, Cd, Pb, Ni and Cr, respectively. Thus, all the heavy metals were within the MPL.

### 2.4. Experimental Design and Treatments

The experiment was conducted in a randomized block design with different recommended doses of fertilizers (RDF), i.e., 150 (N), 75 (P_2_O_5_) and 75 (K_2_O) kg ha^−1^ for rice, and 120 (N), 60 (P_2_O_5_) and 60 (K_2_O) kg ha^−1^ for wheat, and SSL levels which were replicated in triplicate. Treatments in the present study were as follows: T_0_: (no NPK or SSL); T_1_ 100% RDF, T_2_ (T_1_ + SSL 20 Mg ha^−1^); T_3_ (T_1_ + SSL 30 Mg ha^−1^); T_4_ (50% RDF + SSL 20 Mg ha^−1^); T_5_ (60% RDF + SSL 20 Mg ha^−1^); T_6_ (70% RDF + SSL 20 Mg ha^−1^); T_7_ (50% RDF + SSL 30 Mg ha^−1^); T_8_ (60% RDF + SSL 30 Mg ha^−1^) and T_9_ (70% RDF + SSL 30 Mg ha^−1^). A half dose of N and a full dose of P_2_O_5_ and K_2_O were applied at the time of transplanting/sowing of the crops, while the remaining N fertilizer was applied in two equal parts at 30 and 60 days after transplanting or days after sowing (DAT/DAS). The RDF was applied in both the crop and season as per the mentioned treatments. However, SSL (dry weight basis) was applied only once and was spread in the various plots as per treatments and thoroughly mixed with soil one week before the start of the third cycle of rice-wheat.

### 2.5. Data and Collection Procedures

Randomly, five plants from each plot were selected and labelled. The height of both rice and wheat plants was measured using a meter-rod from the base to the tip of the topmost leaf of the plant at 30, 60, and 90 DAT/DAS and the harvest stage, and then averaged. The leaf greenness of the plants (SPAD chlorophyll value) was measured at 30, 60, and 90 DAT/DAS in the uppermost fully expanded leaf using a SPAD-502 (SPAD-502 Plus Konica Minolta). Five representative panicles from rice and the ear from wheat were sampled and grain number in each was recorded. Length (cm) of the panicle/ear was measured from the base of the topmost spikelet. From the yield of the net plot of each experimental unit, 1000 grains were counted and their weight was recorded. The harvest index (HI) was calculated using the following formula:

$$\mathrm{Harvest}\;\mathrm{Index}=\frac{\mathrm{Grain}\;\mathrm{yield}\;(\mathrm{kg}\;{\mathrm{ha}}^{-1})}{\mathrm{Biological}\;\mathrm{yield}\;(\mathrm{kg}\;{\mathrm{ha}}^{-1})}\times 100$$

### 2.6. Plant, Soil and Sewage Sludge Analyses

Rice and wheat grain samples were washed sequentially in detergent solution (0.2% liquid), 0.1 N HCl solution and deionized water then dried at 65 °C until a constant was weight achieved. Nitrogen concentration was determined by digestion (H_2_SO_4_), distillation and a titrimetric method using a standard Kjeldahl Auto analyzer (DISTYL-EM; Pelican, CIT Nagar, Chennai, Tamil Nadu) procedure [29]. Grain samples were digested in a di-acid mixture (HNO_3_:HClO_4_::3:1 *v*/*v*) for the estimation of Cd, Cr, Ni and Pb using an atomic absorption spectrophotometer (Agilent FS-240, 5301 Stevens Creek Blvd, Santa Clara, CA, USA) as per the procedure outlined by [30].After processing, soil samples were analyzed for soil reaction (pH) and electrical conductivity (EC) [31], organic carbon [32], available nitrogen by the alkaline potassium permanganate method [33], available phosphorus by spectrophotometry [34], available potassium by flame photometry [35], and available sulphur by a turbidimetric method [36], and DTPA extractable Zn, Cu, Mn, Fe, Pb, Cd, Cr, and Ni [37] were analyzed by atomic absorption spectrophotometry (AAS) (Agilent FS-240). Total N, P and K content in SSL was analyzed by the methods outlined by [38]. Total heavy metals (Cd, Cr, Ni and Pb) in SSL were analyzed by an aqua regia digestion procedure which consist of digesting SSL samples digested on a hot plate with a mixture of HCl and HNO_3_ (3:1 *v*/*v*) [39] followed by analysis using AAS (Agilent FS-240) as per the procedure outlined by [40]. After completion of the experiment (IV-wheat), total heavy metal (Pb, Cd, Cr, and Ni) content in post-harvest soil was determined by AAS using aqua regia (HCl:HNO_3_::3:1 *v*/*v*) digestion [39]. The certified reference standards (CRS) for Pd (5190–8287), Cd (5190–8270), Cr (5190–8275), and Ni (5190–8298) were purchased from Agilent, 5301 Stevens Creek Blvd, Santa Clara, CA 95051, USA. To control analytical precision, quality control check samples were taken from materials with parameters of known value and set at concentrations near the midpoint of the calibration range. The recovery rate of Pd, Cd, Cr, and Ni were 97.2, 98.5, 96.4, and 98.8%, respectively.

The protein content in grain was calculated by multiplying N (%) in the grain of rice and wheat by a factor of 6.25 [41].

### 2.7. Statistical Data Analysis

The data were statistically analysed using one-way analysis of variance (ANOVA) in SPSS Inc., Chicago Ver. 22. Duncan’s multiple range test (DMRT) was used to test the significance of the difference between the treatments at the 5% level. Figures were drawn using Sigma plot 12.5 software and Microsoft Excel 2016.

## 3. Results and Discussion

### 3.1. Effect of Joint Application of Sewage Sludge and Fertilizers on the Growth of Rice and Wheat

The effect of joint application of SSL with chemical fertilizer (CF) at different growth stages (30, 60, 90 DAT/DAS and harvest stage) of rice and wheat crop is presented in Table 1 and Table 2. The greatest plant height was measured in treatment T_3_, i.e., the combination of 30 Mg ha^−1^ SSL + 100% RDF (104.22 cm) followed by T_2_, i.e., 20 Mg ha^−1^ SSL + 100% RDF (102.41 cm) at the harvest stage of III-rice and these treatments having significantly increased height compared to 100% RDF. However, treatment T_2_ i.e., 20 Mg ha^−1^ SSL + 100% RDF and T_3_ i.e., 30 Mg ha^−1^ SSL + 100% RDF were found to be statistically similar with respect to plant height at the harvest stage. A similar trend was noticed in III-wheat. During 2018–2019, the maximum plant height for IV-rice (102.32) and IV-wheat (103.80) at harvest stage was recorded in treatments T_3_ (30 Mg ha^−1^ SSL + 100% RDF), significantly greater than T_1_ (100% RDF). However, T_3_ (30 Mg ha^−1^ SSL + 100% RDF) was statistically at par with treatment T_2_ (20 Mg ha^−1^ SSL + 100% RDF) in IV-rice and IV-wheat. In IV-wheat crop, application of 20 Mg ha^−1^ SSL + 100% RDF (T_2_) and 30 Mg ha^−1^ SSL + 100% RDF (T_3_) significantly increased plant height over 100% RDF (Table 1 and Table 2).

There was a significant reduction in plant height in T_4_ (50% RDF + 20 Mg ha^−1^ SSL) and T_5_ (60% RDF + 20 Mg ha^−1^ SSL), whereas T_6_ (70% RDF + 20 Mg ha^−1^ SSL), T_7_ (50% RDF + 30 Mg ha^−1^ SSL), T_8_ (60% RDF + 30 Mg ha^−1^ SSL) and T_9_ (70% RDF + 30 Mg ha^−1^ SSL) were at par with T_1_ (100% RDF). The plant heights in treatment T_2_ (100% RDF + 20 Mg ha^−1^ SSL) and T_3_ (100% RDF + 30 Mg ha^−1^ SSL) at harvest were 13.62 and 15.63% higher than T_1_ (100% RDF) in III-rice crop, and in IV-rice the same treatment had respective increases by 10.70 and 14.08% over T_1_ (100% RDF).

In the case of III-wheat, the respective increases in T_2_ (100% RDF + 20 Mg ha^−1^ SSL) and T_3_ (100% RDF + 30 Mg ha^−1^ SSL) were 11.82 and 15.09%, and for IV-wheat, 8.94 and 11.90% over T_1_ (100% RDF). It was observed that at all the growth stages (30, 60 and 90 DAT/DAS), T_3_ (30 Mg ha^−1^ SSL + 100% RDF) and T_2_ (20 Mg ha^−1^ SSL + 100% RDF) showed the highest plant heights in rice and wheat crops (Table 1 and Table 2).

It is well-known that applying SSL with CF to croplands can enhance plant height. According to Latare et al. [21] combining SSL with chemical fertilizers may improve soil fertility and increase the availability of nitrogen and trace elements to plants, thus indirectly enhanced plant development. The addition of SSL with CF enhances the direct availability of N and P from chemical fertilizers, and indirect or slow-release from SSL, which results in increased leaf area and higher dry matter accumulation [42,43]. Thus, the improvement of soil fertility associated with the application of SSL and CF would have supported improved rice and wheat plant growth. Similarly, Zhang et al. [44] revealed a significant increase in rice plant height by greater soil fertility and nutrient status after applying SSL amendments. Our findings also resemble the work of Rehman and Qayyum [45], who reported a significant influence of SSL compost on crop productivity and biomass accumulation in rice and wheat crops.

### 3.2. Leaf Greenness (SPAD) at Different Growth Stages of Rice and Wheat

**Figure 3:**
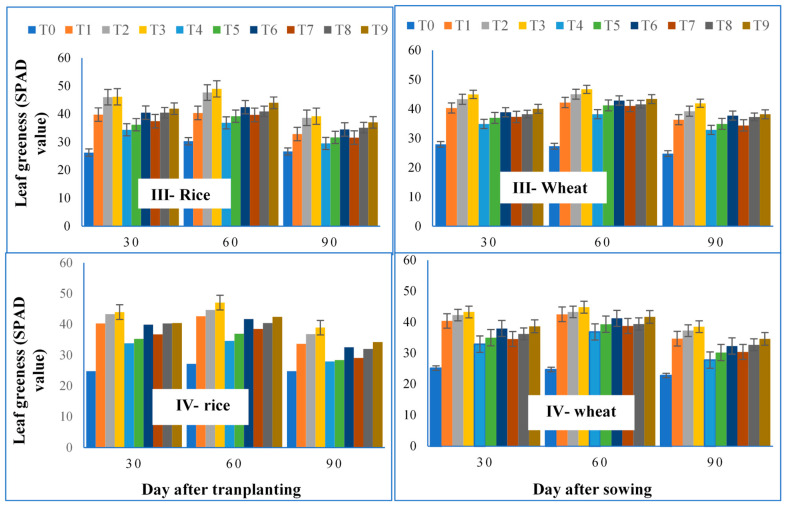
Impact of sewage sludge use with fertilizer on leaf greenness (SPAD value). The bars represent the means ± SE of three replicates.

The data presented in Figure 3 show a significant increase of leaf greenness (chlorophyll content) in rice and wheat due to the joint application of SSL and CF in both years.

Maximum leaf greenness was measured in T_3_ (100% RDF + 30 Mg ha^−1^ SSL) followed by T_2_ (100% RDF + 20 Mg ha^−1^ SSL), and the minimum was in T_0_ (without SSL and CF). Treatment T_3_ (100% RDF + 30 Mg ha^−1^ SSL) and T_2_ (100% RDF + 20 Mg ha^−1^ SSL) had significantly increased leaf greenness over T_1_ (100% RDF) in III-rice, whereas these treatments were statistically similar with 100% RDF (T_1_) in III-Wheat.

However, the treatments from T_4_ toT_9_ showed a non-significant difference of T_1_ in terms of leaf greenness in III-rice and III-wheat in the year 2017–2018. Similarly, the treatments T_2_ (100% RDF + 20 Mg ha^−1^ SSL) and T_3_ (100% RDF + 30 Mg ha^−1^ SSL) were found statistically at par with each other in III-rice and III-wheat. During 2018–2019, the leaf greenness ranged from 24.84 to 44.01 and 25.13 to 43.27 SPAD in IV-Rice and IV-Wheat, respectively. The maximum leaf greenness in IV-Rice and IV-wheat, during both years was seen in T_3_, i.e., the combination of 30 Mg ha^−1^ SSL + 100% RDF at 30 DAT/DAS.

Although, it was noticed that the T_3_ (100% RDF + 30 Mg ha^−1^ SSL), treatment was statistically at par with T_1_ (100% RDF), T_2_ (100% RDF + 20 Mg ha^−1^ SSL), T_6_ (70% RDF + 20 Mg ha^−1^ SSL), T_8_ (60% RDF + 30 Mg ha^−1^ SSL), and T_9_ (100% RDF + 30 Mg ha^−1^ SSL), it was significantly superior over the rest of the treatments in terms of leaf greenness in IV-rice and IV-wheat at 30 DAT during the year 2018–2019. An almost similar trend was observed with the leaf greenness recorded at 60 and 90 DAT/DAS. AT 60 DAT/DAS, a slight increase in plant leaf greenness was noticed compared to observations at 30 DAT/DAS, whereas at 90 DAT, a decrease was noticed compared to 30 and 60 DAT/DAS. Chlorophyll content (SPAD) directly influences the photosynthetic rate of plants. The increase in assimilatory pigments content in leaves was observed when crops were grown in SSL-amended soil. Romani and Beltarre [46] found that repeated 7 years of treatment with SSL (3.7 Mg ha^−1^) resulted in a significant increase in chlorophyll content (SPAD index). Latare et al. [43] reported that at 30 days after transplanting and sowing (DAT/DAS) in rice and wheat, leaf greenness index did not increase but increased significantly at 60 and 90 DAT/DAS. This might be because Fe, Mg, and Mn contents in the SSL, are liberated after decomposition of SSL and remain directly associated with chlorophyll synthesis [47,48].

### 3.3. Effect of Sewage Sludge and Fertilizers on Yield Attributes of Rice and Wheat

The panicle/ear length (cm) ranged between 18.26–37.61 and 12.99–35.95 with mean values of 29.49 and 27.15 in III-Rice and IV-rice, respectively whereas the corresponding value in III-wheat and IV-wheat varied between 6.09–15.54 and 5.90–15.16 with a mean value of 11.89 and 11.28 (Table 3). It was observed that for the rice crop, the significantly highest length of the panicle (37.61 and 35.95 cm) was recorded with T_3_ (100% RDF + 30 Mg ha^−1^ SSL) followed by T_2_ (34.61 and 32.87 cm) during both years, respectively. An almost similar trend was noticed during both years of wheat experimentation. In III-Rice, a significant increase was recorded with T_2_ (18.16%) and T_3_ (28.41%) concerning panicle length. Similarly, the ear length of the wheat crop (III and IV) increased significantly in T_3_ (29.50 and 20.03%) over 100% RDF (Table 3). The result show that the application of SSL with CF increased ear/panicle length. This is due to the role of N in flowering, fruiting, and crop maturation, as well as seed formation. Latare et al. and Jamil et al. [21,49] reported an increase in spike length of wheat with different doses of SSL compared to the non-treated plot. A similar trend was observed in the wheat crop [50]. Zhang et al. [51] reinforced the results, finding that an adequate supply of organic wastes along with NPK fertilizer improves the yield attribute. Thus, combined application of SSL with chemical fertilizer in different levels appears beneficial with respect to yield attributes without showing any toxic effects on plants. The number of grains per panicle/ear (Table 3) varied from 54.44 to 154 and 15.36 to 41.02 in III-rice and III-wheat (2017–2018), respectively. Application of 30 Mg ha^−1^ SSL + 100% RDF resulted in the highest number of grains per panicle i.e., 154.41 and 41.02 in III-rice and III-wheat, respectively.

During 2018–2019, the maximum grains per panicle/ear for IV-rice (146.147) and IV-wheat (39.64) were measured in treatments T_3_ (30 Mg ha^−1^ SSL + 100% RDF). This was significantly superior to T_1_ in IV-rice but statistically similar in IV-wheat (Table 3). A significant reduction was noticed in grains per panicle in T_4_ (50% RDF + 20 Mg ha^−1^ SSL) and T_5_ (60% RDF + 20 Mg ha^−1^ SSL), whereas T_6_ (70% RDF + 20 Mg ha^−1^ SSL), T_7_ (50% RDF + 30 Mg ha^−1^ SSL), T_8_ (60% RDF + 30 Mg ha^−1^ SSL), and T_9_ (70% RDF + 30 Mg ha^−1^ SSL) were at par with T_1_ (100% RDF) in IV-rice. While, IV-wheat showed a marked reduction in T_4_ (50% RDF + 20 Mg ha^−1^ SSL) and T_5_ (60% RDF + 20 Mg ha^−1^ SSL), T_6_ (70% RDF + 20 Mg ha^−1^ SSL), T_7_ (60% RDF + 30 Mg ha^−1^ SSL), T_8_ (70% RDF + 30 Mg ha^−1^ SSL), and T_9_ (70% RDF + 30 Mg ha^−1^ SSL) were statistically at par with respect to grains per panicle. T_2_ (RDF 100% + SSL 20 Mg ha^−1^) was 22.06% higher than T_1_ (RDF 100%) in the III-rice crop, and in the case of IV-rice, the same treatment showed a 12.04% increase over T_1_ (RDF 100%).

**Figure 4:**
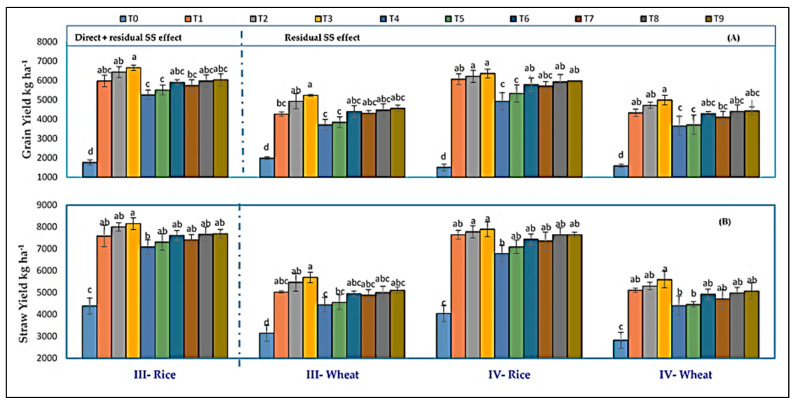
Impact of sewage sludge use with fertilizer on grain yield (**A**) and straw yield (**B**). Data (mean ± SE) followed by the same letter differ non-significantly (*p* ≤ 0.05), while different letters indicate a significant difference (*p* ≤ 0.05).

In the case of III-wheat, T_2_ was 16.81% greater than T_1_ (100% RDF), whereas this increase was only 4.93% in the IV-wheat crop. Tamrabet et al. [52] found an increased number of grains spike^−1^ of wheat after treatment with 20, 30, and 40 Mg ha^−1^ SSL. Moreover, SSL seemed to be more beneficial l the crop than inorganic fertilizer. The SSL treatment statistically improved spike fertility and plant biomass at the heading and maturity stage. By applying SSL and fertilizer, the yield of both crops was significantly improved significantly in all the treatments compared to no fertilizer (Figure 4).

In both years, in rice and wheat crops, a significantly higher grain yield was documented in T_3_, with the combination of 30 Mg ha^−1^ SSL + 100% RDF, followed by T_2_ with 20 Mg ha^−1^ SSL + 100% RDF, compared to other treatments, whereas the lowest yield was recorded in WF (T_0_).

There was no significant difference within the rest of the treatments except T_4_ (50% RDF + 20 Mg ha^−1^ SSL) and T_5_ (60% RDF + 20 Mg ha^−1^ SSL) compared to only 100% RDF (T_1_) in IV-rice and IV-Wheat. The grain yield in treatment T_2_ (100% RDF + 20 Mg ha^−1^ SSL) and T_3_ (100% RDF + 30 Mg ha^−1^ SSL) of III-rice was higher by 7.75% and 11.42%, respectively, compared to treatment T_1_ where 100% RDF was applied. In the case of IV-rice, the grain yield of treatments T_2_ (100% RDF + 20 Mg ha^−1^ SSL) and T_3_ (100% RDF + 30 Mg ha^−1^ SSL) was higher by 2.44% and 4.83%, respectively, than T_1_ (100% RDF). With III-wheat, the grain yield of treatment T_2_ (20 Mg ha^−1^ SSL + 100% RDF) and T_3_ (30 Mg ha^−1^ SSL + 100% RDF) showed a respective increase of 15.55% and 22.75% over T_1_ (100% RDF), whereas, in the case of IV-wheat, treatment T_2_ (20 Mg ha^−1^ SSL + 100% RDF) and T_3_ (30 Mg ha^−1^ SSL + 100% RDF) showed only 9.12% and 15.20% yield increment over T_1_ (100% RDF).

During 2017–2018, in III-rice, application of 100% RDF resulted in statistically similar grain yield in all other treatments except T_0_ (WF). However, the yield of treatment T_1_, i.e., 100% RDF, was at par with 20 Mg ha^−1^ SSL when applied with reduced doses of CF (T_4_, T_5_ and T_6_), and also with 30 Mg ha^−1^ SSL with a reduced dose of CF (T_7_, T_8_ and T_9_). This provides the option of reducing the dose of RDF up to 50% when applied with SSL. A similar trend was observed for the grain yield of III–wheat. During 2018–2019, grain yield of IV-Rice in T_1_ (100% RDF) was statistically similar to T_2_ (20 Mg ha^−1^ SSL + 100% RDF), T_3_ (30 Mg ha^−1^ SSL + 100% RDF), T_6_ (20 Mg ha^−1^ SSL + 70% RDF), T_7_ (30 Mg ha^−1^ SSL + 50% RDF), T_8_ (30 Mg ha^−1^ SSL + 60% RDF) and T_9_ (30 Mg ha^−1^ SSL + 70% RDF). However, a significant reduction in grain yield was noticed in T_4_ (20 Mg ha^−1^ SSL + 50% RDF) and T_5_ (20 Mg ha^−1^ SSL + 60% RDF). An almost similar yield trend was observed for IV wheat. It is evident that providing only 50% RDF with 20 Mg ha^−1^ SSL resulted in yields similar to 100% RDF for the first two crops. However, in subsequent years, i.e., IV-rice and IV-wheat, due to a decrease in the residual effect of SSL (applied in III-rice), the amount of chemical fertilizer had to be increased to 70% RDF with SSL (20 Mg ha^−1^ SSL + 70% RDF) to obtain similar grain yield to that of 100% RDF. There is a strong relationship between yield attributes and yield, particularly with respect to grain number in the panicle/ear. It was noted that the joint application of SSL with chemical fertilizer treatments increased different yield indicators, such as effective tillers and the weight of 1000 grains, thus producing higher grain yield (Figure 4). It was found that the use of SSL in RWCS had the potential to substitute half the dose of fertilizers. The yield increment could be explained by the fact that SSL as a source of organic matter contains various nutrients (macro and micro) and provides them to crops slowly after their decomposition [53]. Thus, improved number of grains per panicle/ear, panicle/ear length, and tillers of rice and wheat were positively correlated with joint application of SSL and chemical fertilizer during both years. The results of the present study are supported by Rehman and Qayyum [45], who noted that SSL treatment increased the growth and yield of rice and wheat, which might be due to higher uptake of water and nutrients by plants.

Data depicted in Figure 4 show that joint application of chemical fertilizer and SSL produced significantly higher straw yield than without fertilization (T_0_) but was at par with T_1_ (100% RDF) in both years. Among all the treatments, T_3_ (100% RDF + 30 Mg ha^−1^ SSL) had the highest straw yield of the rice crop (8150 and 7896 kg ha^−1^) and wheat crop (5695 and 5599 kg ha^−1^), respectively, during the course of the experiments. In III-rice, the straw yield of treatments T_2_ (100% RDF + 20 Mg ha^−1^ SSL), T_3_ (100% RDF + 30 Mg ha^−1^ SSL), and T_9_ (70% RDF + 30 Mg ha^−1^ SSL) increased by 4.07, 5.98, and 0.07%, respectively, compared to treatment T_1_ where 100% RDF was applied. In the case of IV-rice, treatment T_2_ (100% RDF + 20 Mg ha^−1^ SSL) and T_3_ (100% RDF + 30 Mg ha^−1^ SSL) produced 1.10 and 2.64% higher straw yield compared to the T_1_. With the III-wheat crop, straw yield of treatment T_2_ (20 Mg ha^−1^ SSL + 100% RDF), T_3_ (30 Mg ha^−1^ SSL + 100% RDF) and T_9_ (30 Mg ha^−1^ SSL + 70% RDF) showed 8.83, 13.18 and 1.58% increments over 100% RDF (T_1_), whereas, in the case of IV-wheat, only treatment T_2_ (20 Mg ha^−1^ SSL + 100% RDF) and T_3_ (30 Mg ha^−1^ SSL + 100% RDF) showed a positive increment in straw yield over 100% RDF (T_1_). Greater leaf chlorophyll contents improve photosynthetic rate, which results in higher crop biomass and yield. The joint application of SSL with chemical fertilizer improves nutrient availability to the plants which improves their root development, the number of tillers, leaves count and ultimately higher straw production. Similar results were reported by Jamil et al. [49] and Al-Mustafa et al. [54].

**Figure 5:**
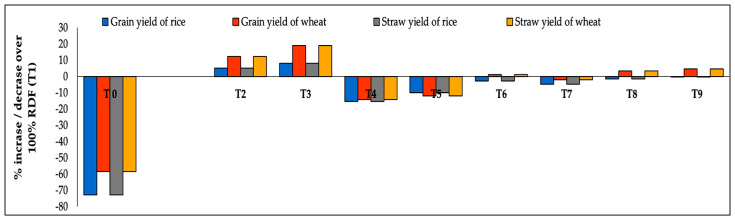
Impact of sewage sludge use with fertilizers on percent increase or decrease in grain and straw yield of rice and wheat (two years of pooled data).

From two years of pooled experimental data, it was found that grain and straw yield of rice and wheat considerably increased or decreased compared to 100% RDF (T_1_) (Figure 5). Application of 30 Mg ha^−1^ SSL + 100% RDF (T_3_) had maximum enhancement of grain yield in rice (8.1%) over 100% RDF (T_1_) followed by the 20 Mg ha^−1^ SSL + 100% RDF plot (5.1%). Application of a reduced dose of CF i.e., 50, 60, and 70% of RDF, along with 20 or 30 Mg ha^−1^ SSL i.e., T_4_ (50% RDF + 20 Mg ha^−1^ SSL), T_5_ (60% RDF + 20 Mg ha^−1^ SSL), T_6_ (70% RDF + 20 Mg ha^−1^ SSL), T_7_ (50% RDF + 30 Mg ha^−1^ SSL), T_8_ (60% RDF + 30 Mg ha^−1^ SSL) and T_9_ (70% RDF + 30 Mg ha^−1^ SSL) resulted in 15, 10, 2.9, 4.9 1.6 and 0.3% reductions in rice grain yield over T_1_, respectively. However, the greatest decrease was seen in the T_0_ treatment (72.9%).

Concerning wheat, the highest increase in grain production over 100% RDF (T_1_) was recorded in T_3_ (30 Mg ha^−1^ SSL + 100% RDF; 18.9%) followed by 20 Mg ha^−1^ SSL + 100% RDF (T_2_), 20 Mg ha^−1^ SSL + 70% RDF (T_6_), 30 Mg ha^−1^ SSL + 60% RDF (T_8_; 3.3%) and 30 Mg ha^−1^ SSL + 70% RDF (T_9_; 4.5%). An almost parallel trend was recorded with respect to the straw yield of rice and wheat. The highest increment in straw yield of rice (4.51%) and wheat (11.3%) over T_1_ (100% RDF).was seen in T_3_ (30 Mg ha^−1^ SSL + 100% RDF). Application of SSL with CF increased the grain and straw of rice and wheat yield percentage compared to 100% RDF due to slow release of nutrients from SSL throughout the period of crop growth, this finding being in accordance with the results of [22]. Application of any kind of fertilizer resulted in a greater response in wheat crop compared to rice due to different cultivation practices of rice and wheat. Yadav et al. [55], Gami et al. [56] and Bhatt et al. [57] stated that long-term integrated use of manure and fertilizers practiced in RWCS produces higher wheat yield than rice over only chemically fertilized plots.

### 3.4. Sewage Sludge and Fertilizers Influence the Harvest Index and Test Weight of Rice and Wheat

Data pertaining to HI as affected by the joint application of SSL and fertilizer as shown in Table 4. Among all the treatments, T_3_ (100% RDF + 30 Mg ha^−1^ SSL) produced the highest HI in rice (45.00 and 44.67) and wheat crops (47.97 and 47.19) during both years. Significantly lower HI was recorded in T_0_ in the rice crop (28.83 and 27.15) and in the wheat crop (38.66 and 36.12) during both years. Latare et al. [21], stated that an SSL treatment improved HI significantly in wheat but was non-significant in rice. This might be due to additional nutrients available to residual grown wheat from SSL-altered soil.

The data with respect to 1000 grain weight (test weight) of rice and wheat are shown in Table 4. Among all the treatments, T_3_ (100% RDF + 30 Mg ha^−1^ SSL) had the highest test weight in the rice (25.88 and 25.80) and wheat crop (33.86 and 33.18) during 2017–2018 and 2018–2019, respectively. In the III-Rice crop, T_2_ (100% RDF + 20 Mg ha^−1^ SSL) and T_3_ (100% RDF + 30 Mg ha^−1^ SSL) had 16.61% and 18.97% greater test weights in comparison to T_1_ (100% RDF). In the case of the IV-rice crop, T_2_ (100% RDF + 20 Mg ha^−1^ SSL) and T_3_ (100% RDF + 30 Mg ha^−1^ SSL) produced, respectively, 12.41% and 13.54% higher test weights over T_1_ (100% RDF), whereas, in the case of the III-wheat crop, the test weight in T_2_ (100% RDF + 20 Mg ha^−1^ SSL) and T_3_ (100% RDF + 30 Mg ha^−1^ SSL) was 9.10% and 12.99% higher than T_1_ (100% RDF), respectively. In the IV-wheat crop, T_2_ (20 Mg ha^−1^ SSL + 100% RDF) and T_3_ (30 Mg ha^−1^ SSL + 60% RDF) showed 2.50 and 6.61% higher test weights over 100% RDF (T_1_), respectively. The SSL-amended soil favoured vegetative growth, development, and maturity of rice and wheat. This can be explained by optimal uptake of trace, micronutrients, and macronutrients by crop plants that support various biochemical and physiological processes, resulting in higher vigour of seeds. Jamil et al. [49] reported a significant rise in 1000 grain weight of wheat with the use of SSL compared to control. Barbarick et al. [58] and Elsokkary et al. [59] also reported that SSL-based nutrient management improved the productivity of crop plants, including 1000 grain weight.

### 3.5. Sewage Sludge and Fertilizers Influence the Nitrogen (%) and Protein (%) of Rice and Wheat

Application of SSL and fertilizer significantly influenced the nitrogen concentration and protein content of rice and wheat crops during both years (Table 5). 

A significantly higher concentration of N and protein content of rice (1.78 and 1.66%, 11.15 and 10.35%) and wheat (2.69 and 2.66, 16.81 and 14.13%) were recorded with 100% RDF + 30 Mg ha^−1^ SSL during 2017–2018 and 2018–2019, respectively, whereas the lowest was recorded in the non-fertilized plot. The treatments (T_4_–T_9_), which received a reduced dose of CF with SSL, were statistically at par with 100% RDF up to IV-Rice but in the case of IV-wheat, a significant reduction was noticed with treatments T_4_ (50% RDF + 20 Mg ha^−1^ SSL) and T_5_ (60% RDF + 20 Mg ha^−1^ SSL) with respect to N concentration (Table 5).

Protein content in treatments that received a reduced dose of CF with SSL, i.e., T_4_ to T_9_, were statistically similar to the 100% RDF treatment during the study. The addition of SSL with CF increased the grain N concentration, because both are a good source of N and resulted in a greater supply of N to the plant [60]. This effect was observed in SSL and RDF-fertilized plots in comparison to nonfertilized plots. The N in the soil helps the decomposition process and, as a result, the rate of decomposition in RDF with sludge-supplemented soils may be greater, leading to quicker SSL breakdown. Nitrogen is required for the synthesis of amino acids and increases the protein content of cereals considerably [61,62]. Yamur et al. [63] stated that SSL application improved protein content from 19.82 to 23.92% in lentils.

### 3.6. Sewage Sludge and Fertilizers Influence the Cadmium, Chromium, Nickel and Lead Concentration (mg kg^−1^) of Rice and Wheat

The SSL and fertilizer treatments had higher Cd concentration in rice and wheat grain compared to 100% RDF (Table 6) treatment. In III-Rice, the highest Cd concentration was observed in treatment T_3_ (100% RDF + 30 Mg ha^−1^ SSL) at 1.27 mg kg^−1^, followed by T_9_ (70% RDF + 30 Mg ha^−1^ SSL) at 1.19 mg kg^−1^, and T_8_ 60% (RDF + 30 Mg ha^−1^ SSL) at 1.14 mg kg^−1^) which showed 184, 166 and 154% increases over T_1_ (100% RDF). An almost similar trend was seen in III-Wheat and the highest Cd concentration was observed in treatment T_3_ (100% RDF + 30 Mg ha^−1^ SSL) at 1.36 mg kg^−1^, followed by T_9_ (70% RDF + 30 Mg ha^−1^ SSL) at 1.26 mg kg^−1^, and T_8_ (60% RDF + 30 Mg ha^−1^ SSL) at 1.25 mg kg^−1^, which were 167, 146 and 144% higher that T_1_ (0.51 mg kg^−1^). The lowest Cd concentration was seen in T_0_ (0.33 mg kg^−1^) which was statistically lower than T_1_ (100% RDF).

During the 2018–2019 (IV-rice and IV-Wheat) season of the experiment, treatments receiving SSL with CF had significantly higher Cd concentrations then T_1_ in grains of IV-rice, and a similar trend was noticed in IV-wheat. In IV-Rice, the Cd concentration was in the order of: T_3_ (1.23 mg kg^−1^) > T_9_ (1.16 mg kg^−1^) > T_8_ (1.11 mg kg^−1^) > T_2_ (1.10 mg kg^−1^) > T_7_ (1.05 mg kg^−1^) > T_6_ (0.83 mg kg^−1^) = T_5_ (0.83 mg kg^−1^) > T_4_ (0.80 mg kg^−1^) > T_1_ (0.42 mg kg^−1^) > T_0_ (0.25 mg kg^−1^), with respective increases of 195, 178, 166, 163, 153, 99, 99 and 93% over T_1_ (0.42 mg kg^−1^). In IV-Wheat, the order was: T_3_ (167%) > T_9_ (150%) > T_8_ (148%) > T_2_ (145%) > T_7_ (141%) > T_6_ (88%) > T_5_ (85%) >T_4_ (74% increase) over T_1_ (100% RDF). Overall, the Cd concentration in grain increased with joint application of sewage sludge, i.e., 20 and 30 Mg ha^−1^, and full or reduced dose of CF, compared to 100% RDF. This study revealed that Cd concentration was highest (1.36 mg kg^−1^) in wheat grain but below the permissible limits of Cd, which are 4 mg kg^−1^ and 12 mg kg^−1^ in Poland and the Czech Republic, respectively [64]. Furthermore, the study also found that Cd accumulation in grain did not exceed the phytotoxic threshold level of 5–30 mg kg^−1^ [65]. The Cd concentration was low in rice compared to wheat in the grain. In comparison to wheat, the lower Cd content in rice could be explained due to the submergence condition. Under flooded condition, Cd forms insoluble compounds such as Cd sulfide and/or Cd carbonate which reduce its availability to the rice plants [66,67]. Greger [68], reported that magnification of heavy metals levels in plants is influenced by soil properties, SSL composition, the application rate of SSL, and elemental speciation [69].

The data pertaining to Cr concentration in grain (Table 6) showed significantly higher Cr concentration with all combined treatments of SSL with CF compared to RDF. During 2017–2018, the maximum Cr concentration in III-rice was recorded in treatment T_3_ (4.90) followed by T_9_ (4.85) and T_8_ (4.79), whereas in III-Wheat it was in treatment T_3_ (4.49) followed by T_9_ (4.34) and T_2_ (4.22), with a respective significant increase of 148, 146 and 143% in III-Rice and 156, 148 and 141% in III-wheat compared to 100% RDF (T_1_). During 2018–2019 (IV-rice and IV-Wheat), the maximum Cr concentration in IV-Rice (4.11 mg kg^−1^) and IV-wheat (3.55 mg kg^−1^) was recorded in T_3_ (30 Mg ha^−1^ SSL). The application of 20 and 30 Mg ha^−1^ SSL with full or reduced doses of CF (T_2_, T_3_, T_4_, T_5_, T_6_, T_7_, T_8_ and T_9_) significantly increased the Cr concentration in grain by 2.43, 2.86, 2.00, 2.06, 2.11, 2.37, 2.46 and 2.76 times in IV-Rice and 2.55, 2.79, 1.96, 1.99, 2.11, 2.24, 2.37 and 2.73 times in IV-Wheat, respectively, over 100% RDF (T_1_). The phytotoxic limit of Cr is 5–30 mg kg^−1^ [65]. This result indicates that Cr concentration in grain was within the acceptable limit and did not cross the phytotoxic threshold level.

The results show that the Ni concentration in III-rice varied from 7.65 to 19.00 mg kg^−1^, whereas, in III-wheat it ranged between 7.93 to 17.31 mg kg^−1^ (Table 7). During 2017–2018, the maximum Ni concentration in III-rice (19.00 mg kg^−1^) and III-wheat (17.31 mg kg^−1^) was recorded in treatment T_3_ (100% RDF + 30 Mg ha^−1^ SSL). The application of 20 and 30 Mg ha^−1^ SSL along with a full dose or reduced dose of CF significantly increased the Ni concentration in grains of III-rice and III-wheat compared to 100% RDF (T_1_). During 2018–2019, the Ni concentration in grain ranged between 7.35 to 5.48, and 6.28 to 14.35 mg kg^−1^, in IV-rice and IV-wheat, respectively. The greatest Ni concentration in IV-Rice (15.48 mg kg^−1^) and IV-wheat (14.35 mg kg^−1^) was recorded in T_3_ (100% RDF + 30 Mg ha^−1^ SSL). The application of 30 Mg ha^−1^ SSL along with a reduced dose of CF (50, 60, 70% of RDF), i.e., T_7_, T_8_ and T_9_ resulted in significant increases in Ni concentration in grains by 45, 48 and 61% in IV-rice, respectively, over 100% RDF (T_1_), whereas in IV-wheat, these treatments were statistically at par with T_1_ (100% RDF). All the treatments amended with 20 Mg ha^−1^ SSL along with a reduced dose of CF (50, 60, 70 of RDF), i.e., T_4_, T_5_ and T_6_, did not show a significant increase of Ni in grain compared to 100% RDF in both crops (IV-rice and IV-wheat). The Ni concentration was below the phytotoxic limit of 10–100 mg kg^−1^ as in [65]. Out of the various treatments (Table 7), T_3_ (100% RDF + 30 Mg ha^−1^ SSL) had the highest Pb concentration in III-rice grain (1.99) and III-wheat grain (1.94). The joint application of 20 and 30 Mg ha^−1^ SSL and full dose or reduced doses CF (T_2_, T_3_, T_4_, T_5_, T_6_, T_7_, T_8_ and T_9_) in III-rice significantly increased the Pb concentration in grain by 1.89, 2.34, 1.52, 1.57, 1.65, 1.77, 1.95 and 2.25 times, respectively, compared to 100% RDF. The corresponding increases of Pb concentration in III-wheat were 2.53, 2.88, 1.83, 1.91, 2.04, 2.08, 2.14 and 2.77 times. 

During 2018–2019, (IV-rice and IV-wheat), all the treatments of SSL (T_3_–T_9_) were significantly higher in Pb concentration compared to 100% RDF. The Pb concentration in grain ranged from 0.40 to 1.81 and 0.37 to 1.53 mg kg^−1^ in IV-rice and IV-wheat, respectively. The highest Pb concentrations in IV-Rice (1.81 mg kg^−1^) and IV-Wheat (1.53 mg kg^−1^) were recorded in T_3_ (30 Mg ha^−1^ SSL). Treatments T_2_, T_3_, T_4_, T_5_, T_6_, T_7_, T_8_ and T_9_ when compared with RDF had corresponding increases of 2.24, 2.87, 1.87, 1.89, 2.03, 2.13 and 2.75 times in IV-rice and 1.95, 2.57, 1.58, 1.67, 1.67, 1.94, 1.96 and 2 times in IV-wheat. The lowest Pb concentration was recorded in T_0_ during the course of the experiment. The present investigation revealed that Pb (highest, 1.99 mg kg^−1^ in III-rice) in rice and wheat grain were below phytotoxic limits (30–300 mg kg^−1^) as outlined by [65]. Singh and Agrawal [70] and Eid et al. [71] reported an increase in heavy metal concentration in the areal parts of barley by application of SSL. Zoubi et al. [72] reported similar findings.

### 3.7. Total Heavy Metal Content in Post-Harvest Soil after Completion of the Experiment

**Figure 6:**
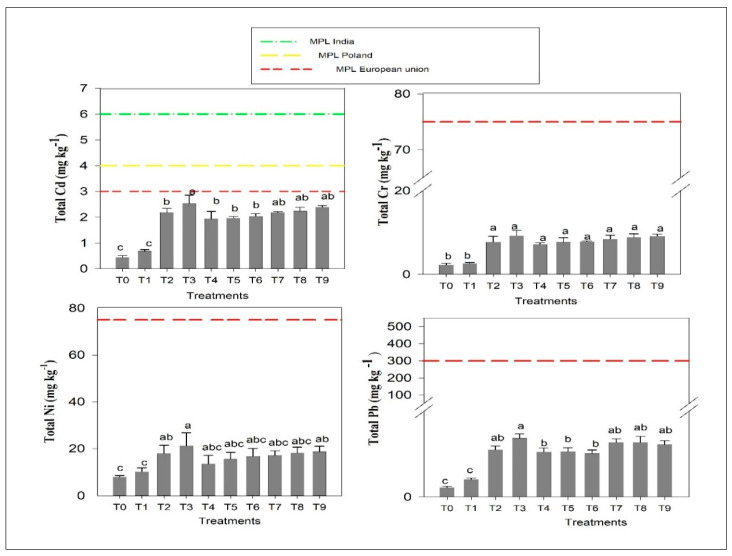
Impact of sewage sludge use with fertilizers on total heavy metals buildup in soils after completion of the experiment. Data (mean ± SE) followed by the same letter differ non-significantly (*p* ≤ 0.05), while different letters indicate a significant difference (*p* ≤ 0.05).

The total Cd, Cr, Ni and Pb content in soils after the termination of the experiment ranged from 0.44 to 2.53, 2.25 to 9.26, 7.98 to 21.23 and 4.45 to 27.36 mg kg^−1^, respectively (Figure 6). Among all the treatments, T_3_ (100% RDF + 30 Mg ha^−1^ SSL) had the highest total Cd, Cr, Ni and Pb after harvest of IV-wheat. The treatments that received 30 SSL Mg ha^−1^ (T_3,_ T_7_, T_8_ and T_9_) increased the total Cd content by 5.75, 3.2 3.3 and 3.6 times, respectively over T_1_, whereas, treatments T_2_, T_4_, T_5_ and T_6_ that received a lower dose (20 SSL Mg ha^−1^) increased by 3.2, 2.9, 2.9 and 3.0 times, respectively. However, total Cr content soil after harvest of the IV-wheat crop showed a declining trend in the order of T_3_ (9.26) > T_9_ (9.15 mg kg^−1^) > T_8_ (8.88 mg kg^−1^) > T_7_ (8.45 mg kg^−1^) > T_2_ (7.77 mg kg^−1^) > T_6_ (7.89 mg kg^−1^) > T_5_ (7.69 mg kg^−1^) > T_4_ (7.22 mg kg^−1^).

These data were statistically similar to one another but significantly greater T_1_ (2.59 mg kg^−1^). The total Ni content in post-harvest soils significantly increased due to the application of SSL. The 100% RDF treatment, i.e., T_1_ (10.25 mg kg^−1^) was statistically at par with all other treatments except T_2_ (18.0 mg kg^−1^), T_3_ (21.23 mg kg^−1^) and T_9_ (18.65 mg kg^−1^) which had the highest content of Ni in the post-harvest soil. Treatments T_3_ (100% RDF + 30 Mg ha^−1^ SSL), T_7_ (50% RDF + 30 Mg ha^−1^ SSL), T_8_ (60% RDF + 30 Mg ha^−1^ SSL) and T_9_ (70% RDF + 30 Mg ha^−1^ SSL) had increased Pb content by 3.32, 3.09 3.08 and 2.98 times, respectively, over T_1_ (100% RDF), whereas in treatments T_2_ (100% RDF + 20 Mg ha^−1^ SSL), T_4_ (50% RDF + 20 Mg ha^−1^ SSL), T_5_ (60% RDF + 20 Mg ha^−1^ SSL) and T_6_ (70% RDF + 20 Mg ha^−1^ SSL) with lower dose of SSL (20 Mg ha^−1^), the respective increases were 2.66, 2.55, 2.58 and 2.45 times. The total heavy metal contents in soil were within the maximum permissible limits (MPL) with respect to the limits [64] for India and for Poland and the European Union [28] (Figure 6). The results indicate that total heavy metal contents in soil were within MPL even after applying the highest dose of SSL (30 Mg ha^−1^) with 100% RDF.

## 4. Conclusions

Application of 20 or 30 Mg ha^−1^ SSL in conjunction with 100% RDF significantly improved the productivity of the rice-wheat system compared to 100% RDF. It was observed that 20 or 30 Mg ha^−1^ SSL along with recommended or reduced doses of fertilizer significantly increased the heavy metal content in grains and experimental soil compared to 100% RDF, or absolute control plots, but this buildup was within permissible limits. Therefore, to obtain optimum grain yield (at par with RDF 100%), it is recommended to apply only a 50% dose of RDF in the first two crops and a 70% dose of RDF in the next two crops with a one-time application of 20 t ha^−1^ SSL. Regular monitoring of heavy metal buildup in soil, and its subsequent increase in the edible part of rice and wheat, needs to be strictly tracked to avoid risks related to soil and human health. However, this study needs to be confirmed by long-term experiments before recommendations for other agro-climatic regions.

## Figures and Tables

**Table 1:** Impact of sewage sludge use with fertilizers on plant height of rice and wheat.

Treatments	Plant Height (cm) at 30 DAT/DAS			
	2017–2018		2018–2019	
	III-Rice	III-Wheat	IV-Rice	IV-Wheat
T_0_ (WF)	45.49 ± 1.44 d	18.75 ± 2.56 c	42.45 ± 2.32 e	16.48 ± 2.44 d
T_1_ (RDF 100)	75.60 ± 0.68 ab	30.05 ± 1.81 ab	76.54 ± 0.94 ab	30.97 ± 0.95 abc
T_2_ (RDF 100 + SSL 20)	79.31 ± 1.51 a	33.17 ± 2.32 ab	78.91 ± 1.70 a	32.11 ± 1.06 ab
T_3_ (RDF 100 + SSL 30)	81.49 ± 2.12 a	36.87 ± 1.53 a	79.85 ± 2.38 a	35.10 ± 1.75 a
T_4_ (RDF 50 + SSL 20)	64.69 ± 0.97 c	26.55 ± 1.50 bc	63.42 ± 1.76 d	24.39 ± 1.73 c
T_5_ (RDF 60 + SSL 20)	67.51 ± 0.90 bc	27.50 ± 1.32 b	65.90 ± 1.20 cd	25.90 ± 1.54 bc
T_6_ (RDF 70 + SSL 20)	70.43 ± 2.53 bc	28.96 ± 0.78 ab	68.25 ± 0.71 bcd	27.30 ± 0.68 bc
T_7_ (RDF 50 + SSL 30)	66.98 ± 0.33 bc	28.21 ± 2.59 b	64.57 ± 0.33 cd	26.78 ± 1.07 bc
T_8_ (RDF 60 + SSL 30)	70.54 ± 2.20 bc	30.76 ± 0.74 ab	69.60 ± 2.23 bcd	29.36 ± 1.32 abc
T_9_ (RDF 70 + SSL 30)	73.80 ± 2.83 ab	31.14 ± 1.47 ab	72.02 ± 1.75 abc	30.38 ± 1.41 abc
Significance level	**	**	**	**
Treatments	Plant Height (cm) at 60 DAT/DAS			
T_0_ (WF)	56.94 ± 2.57 e	36.78 ± 0.51 e	55.57 ± 2.5 e	34.91 ± 1.35 d
T_1_ (RDF 100)	82.28 ± 2.11 bcd	61.38 ± 2.33 cd	83.07 ± 1.96 abcd	62.95 ± 1.22 ab
T_2_ (RDF 100 + SSL 20)	89.63 ± 0.30 ab	70.42 ± 2.03 ab	87.77 ± 2.3 ab	67.891.15 a
T_3_ (RDF 100 + SSL 30)	92.33 ± 1.58 a	73.41 ± 1.99 a	89.62 ± 0.86 a	69.46 ± 1.57 a
T_4_ (RDF 50 + SSL 20)	74.63 ± 2.09 d	56.23 ± 2.54 d	73.54 ± 1.56 d	52.86 ± 2.02 c
T_5_ (RDF 60 + SSL 20)	77.77 ± 0.73 cd	57.67 ± 1.24 cd	77.03 ± 2.10 cd	55.58 ± 1.60 bc
T_6_ (RDF 70 + SSL 20)	79.19 ± 1.79 cd	61.95 ± 2.04 bcd	78.07 ± 1.58 bcd	58.42 ± 1.73 bc
T_7_ (RDF 50 + SSL 30)	78.08 ± 0.58 cd	59.58 ± 1.95 cd	77.02 ± 2.45 cd	57.06 ± 1.45 bc
T_8_ (RDF 60 + SSL 30)	81.03 ± 1.46 bcd	64.09 ± 0.48 bcd	79.98 ± 2.36 abcd	61.27 ± 1.87 abc
T_9_ (RDF 70 + SSL 30)	84.41 ± 2.47 abc	66.26 ± 0.62 abc	84.05 ± 0.98 abc	63.78 ± 2.77 ab
Significance level	**	**	**	**

Mean values within the same column having the same letters differ non-significantly (*p* ≤ 0.01), while different letters indicate significant difference (*p* ≤ 0.01). Mean (±SE) was taken from three replicates for each treatment. **, indicates significant at 1% level of probability.

**Table 2:** Impact of sewage sludge use with fertilizers on plant height of rice and wheat.

Treatments	Plant Height (cm) at 90 DAT/DAS			
	2017–2018		2018–2019	
	III-Rice	III-Wheat	IV-Rice	IV-Wheat
T_0_ (WF)	67.51 ± 2.85 c	62.26 ± 1.13 d	62.58 ± 1.23 d	60.62 ± 2.98 c
T_1_ (RDF 100)	92.28 ± 6.52 b	89.90 ± 0.97 bc	93.84 ± 2.04 bc	92.61 ± 1.26 ab
T_2_ (RDF 100 + SSL 20)	105.47 ± 1.05 a	101.15 ± 2.61 a	104.91 ± 2.66 ab	100.03 ± 4.73 a
T_3_ (RDF 100 + SSL 30)	108.02 ± 1.71 a	103.66 ± 1.63 a	106.87 ± 2.39 a	101.17 ± 1.44 a
T_4_ (RDF 50 + SSL 20)	87.80 ± 0.90 b	85.27 ± 2.31 c	85.84 ± 2.30 c	83.10 ± 1.83 b
T_5_ (RDF 60 + SSL 20)	88.50 ± 0.68 b	87.21 ± 0.05 bc	86.40 ± 2.57 c	84.77 ± 2.47 b
T_6_ (RDF 70 + SSL 20)	90.15 ± 1.3 b	89.09 ± 1.30 bc	88.83 ± 2.35 c	85.90 ± 1.24 b
T_7_ (RDF 50 + SSL 30)	90.95 ± 2.80 b	92.29 ± 0.65 bc	90.11 ± 2.38 c	90.51 ± 1.69 ab
T_8_ (RDF 60 + SSL 30)	96.03 ± 1.39 ab	92.97 ± 0.72 b	95.09 ± b3.02 c	91.26 ± 1.63 ab
T_9_ (RDF 70 + SSL 30)	97.48 ± 0.95 ab	93.75 ± 0.42 b	97.01 ± 1.85 abc	93.31 ± 1.41 ab
Significance level	**	**	**	**
Treatments	Plant Height (cm) at Harvest			
T_0_ (WF)	64.95 ± 0.41 d	61.59 ± 1.98 d	61.83 ± 1.98 c	64.86 ± 3.53 e
T_1_ (RDF 100)	90.13 ± 0.89 bc	89.10 ± 0.81 c	89.69 ± 0.81 b	92.76 ± 1.72 bc
T_2_ (RDF 100 + SSL 20)	102.41 ± 1.94 a	99.64 ± 0.79 ab	99.27 ± 0.79 a	101.06 ± 1.81 a
T_3_ (RDF 100 + SSL 30)	104.22 ± 1.55 a	102.55 ± 2.65 a	102.32 ± 2.65 a	103.80 ± 2.12 a
T_4_ (RDF 50 + SSL 20)	86.30 ± 1.59 c	84.96 ± 2.42 c	83.22 ± 2.24 b	84.61 ± 0.84 d
T_5_ (RDF 60 + SSL 20)	88.08 ± 1.27 c	85.46 ± 3.76 c	84.62 ± 3.73 b	85.79 ± 2.09 d
T_6_ (RDF 70 + SSL 20)	88.95 ± 1.85 bc	88.62 ± 2.98 c	87.23 ± 2.90 b	87.54 ± 1.12 cd
T_7_ (RDF 50 + SSL 30)	89.38 ± 3.37 bc	91.90 ± 3.43 bc	86.40 ± 3.43 b	93.20 ± 2.35 bc
T_8_ (RDF 60 + SSL 30)	93.10 ± 3.74 bc	92.34 ± 1.55 bc	91.01 ± 1.55 b	94.11 ± 2.34 bc
T_9_ (RDF 70 + SSL 30)	95.49 ± 1.84 b	93.22 ± 1.24 bc	91.11 ± 1.24 b	97.18 ± 2.57 ab
Significance level	**	**	**	**

Mean values within the same column having alike alphabets differ non-significantly (*p* ≤ 0.05), while different alphabets show a significant difference (*p* ≤ 0.05). Mean (±SE) was taken from three replicates for each treatment. **, indicates significant at 1% level of probability.

**Table 3:** Impact of sewage sludge use with fertilizers on panicle/ear length and grain per panicle or ear of rice and wheat.

Treatments	Panicle/Ear Length (cm)			
	2017–2018		2018–2019	
	III-Rice	III-Wheat	IV-Rice	IV-Wheat
T_0_ (WF)	18.26 ± 1.34 e	6.09 ± 1.01 c	12.99 ± 2.11 e	5.90 ± 0.54 e
T_1_ (RDF 100)	29.29 ± 1.36 cd	12.00 ± 1.05 b	30.36 ± 2.14 bc	12.63 ± 0.85 bc
T_2_ (RDF 100 + SSL 20)	34.61 ± 1.14 ab	13.24 ± 0.87 ab	32.87 ± 0.77 ab	13.08 ± 0.074 ab
T_3_ (RDF 100 + SSL 30)	37.61 ± 1.98 a	15.54 ± 1.12 a	35.95 ± 0.90 a	15.16 ± 0.62 a
T_4_ (RDF 50 + SSL 20)	25.27 ± 1.83 d	11.22 ± 0.85 b	24.30 ± 1.65 d	9.88 ± 0.55 d
T_5_ (RDF 60 + SSL 20)	27.10 ± 1.63 cd	11.50 ± 0.43 b	26.71 ± 1.35 cd	10.01 ± 0.41 de
T_6_ (RDF 70 + SSL 20)	31.48 ± 1.02 bc	11.97 ± 0.65 b	30.71 ± 1.61 bc	10.64 ± 0.25 cde
T_7_ (RDF 50 + SSL 30)	28.04 ± 1.12 cd	11.45 ± 0.67 b	26.44 ± 1.11 cd	11.05 ± 1.23 bcde
T_8_ (RDF 60 + SSL 30)	31.00 ± 2.83 bc	12.47 ± 0.64 b	29.93 ± 0.52 bc	12.19 ± 1.28 bcde
T_9_ (RDF 70 + SSL 30)	32.25 ± 1.03 bc	12.71 ± 0.26 b	31.43 ± 0.86 b	12.27 ± 0.20 bcd
Significance level	**	**	**	**
Grains/Panicle/Ear				
T_0_ (WF)	54.44 ± 2.80 e	15.36 ± 0.49 f	53.04 ± 3.53 g	14.38 ± 1.07 e
T_1_ (RDF 100)	122.99 ± 8.11 d	33.37 ± 0.63 cde	125.71 ± 5.89 cde	35.79 ± 2.70 ab
T_2_ (RDF 100 + SSL 20)	150.16 ± 3.47 ab	38.98 ± 0.13 ab	140.85 ± 5.32 ab	37.55 ± 1.23 ab
T_3_ (RDF 100 + SSL 30)	154.41 ± 2.23 a	41.02 ± 1.35 a	146.17 ± 3.39 a	39.64 ± 1.44 a
T_4_ (RDF 50 + SSL 20)	114.04 ± 3.30 d	31.73 ± 1.84 e	108.57 ± 4.90 f	28.84 ± 1.29 d
T_5_ (RDF 60 + SSL 20)	120.75 ± 1.06 d	32.48 ± 1.99 de	116.19 e ± 0.51 f	29.73 ± 1.51 cd
T_6_ (RDF 70 + SSL 20)	139.42 ± b2.90 c	35.39 ± 1.03 bcde	130.57 ± 0.45 bcd	32.94 ± 2.26 bcd
T_7_ (RDF 50 + SSL 30)	125.26 ± 2.47 d	33.25 ± 1.83 cde	120.72 ± 2.54 de	30.30 ± 0.37 cd
T_8_ (RDF 60 + SSL 30)	136.84 ± 2.15 c	36.10 ± 0.54 bcd	130.40 ± 3.97 bcd	34.14 ± 0.30 bc
T_9_ (RDF 70 + SSL 30)	142.07 ± 1.55 bc	37.05 ± 1.07 bc	133.27 ± 2.90 bc	34.42 ± 1.24 bc
Significance level	**	**	**	**

Mean values within the same column having the same letter differ non-significantly (*p* ≤ 0.01), while different letters indicate a significant difference (*p* ≤ 0.01). Mean (±SE) was taken from three replicates for each treatment. **, indicates significant at 1% level of probability.

**Table 4:** Impact of sewage sludge use with fertilizers on harvest index and 1000 grain weight.

Treatment	Harvest Index (%)			
	2017–2018		2018–2019	
	III-Rice	III-Wheat	IV-Rice	IV-Wheat
T_0_ (WF)	28.83 ± 2.83 b	38.66 ± 0.76 b	27.15 ± 0.51 b	36.12 ± 1.73 b
T_1_ (RDF 100)	43.83 ± 2.72 a	45.85 ± 1.22 a	44.12 ± 0.53 a	45.86 ± 1.39 ab
T_2_ (RDF 100 + SSL 20)	44.58 ± 0.78 a	47.37 ± 2.67 a	44.44 ± 1.88 a	47.13 ± 0.27 a
T_3_ (RDF 100 + SSL 30)	45.00 ± 0.41 a	47.97 ± 1.25 a	44.67 ± 0.86 a	47.19 ± 1.35 a
T_4_ (RDF 50 + SSL 20)	42.68 ± 0.73 a	45.31 ± 1.60 a	42.09 ± 2.55 a	45.27 ± 3.87 ab
T_5_ (RDF 60 + SSL 20)	43.04 ± 1.77 a	45.82 ± 0.77 a	42.86 ± 0.78 a	45.43 ± 0.27 ab
T_6_ (RDF 70 + SSL 20)	43.74 ± 1.07 a	46.92 ± 0.92 a	43.73 ± 1.45 a	46.61 ± 1.19 a
T_7_ (RDF 50 + SSL 30)	43.63 ± 0.50 a	47.00 ± 1.10 a	43.73 ± 0.34 a	46.66 ± 2.18 a
T_8_ (RDF 60 + SSL 30)	43.83 ± 2.14 a	47.11 ± 0.69 a	43.73 ± 2.06 a	46.86 ± 3.10 a
T_9_ (RDF 70 + SSL 30)	43.93 ± 0.72 a	47.19 ± 0.50 a	43.90 ± 1.10 a	46.94 ± 0.62 a
Significance level	**	**	**	**
	1000 Grain Weight (g)			
T_0_ (WF)	15.62 ± 1.85 d	28.89 ± 0.63 a	12.95 ± 0.34 d	28.20 ± 1.28 a
T_1_ (RDF 100)	21.75 ± 1.50 bc	32.35 ± 0.84 a	21.84 ± 1.11 abc	31.69 ± 0.73 a
T_2_ (RDF 100 + SSL 20)	25.37 ± 0.49 a	32.93 ± 0.62 a	24.55 ± 1.82 ab	32.19 ± 0.55 a
T_3_ (RDF 100 + SSL 30)	25.88 ± 0.24 a	33.86 ± 0.29 a	24.80 ± 1.29 a	33.18 ± 1.75 a
T_4_ (RDF 50 + SSL 20)	20.62 ± 0.50 c	31.23 ± 0.75 a	20.21 ± 0.45 c	30.57 ± 1.08 a
T_5_ (RDF 60 + SSL 20)	20.75 ± 1.20 c	31.23 ± 0.81 a	20.37 ± 0.56 c	30.27 ± 0.83 a
T_6_ (RDF 70 + SSL 20)	22.90 ± 0.49 abc	31.53 ± 0.42 a	21.16 ± 0.81 bc	30.64 ± 0.27 a
T_7_ (RDF 50 + SSL 30)	21.81 ± 1.21 bc	31.53 ± 0.60 a	20.39 ± 1.09 c	30.47 ± 0.10 a
T_8_ (RDF 60 + SSL 30)	24.30 ± 0.26 ab	32.80 ± 1.12 a	22.65 ± 1.14 abc	32.15 ± 1.37 a
T_9_ (RDF 70 + SSL 30)	24.87 ± 0.33 ab	32.41 ± 0.60 a	24.08 ± 1.26 ab	31.84 ± 0.34 a
Significance level	**	**	**	**

Mean values within the same column having the same letters differ non-significantly (*p* ≤ 0.01), while different letters indicate a significant difference (*p* ≤ 0.01). Mean (±SE) was taken from three replicates for each treatment. **, indicates significant at 1% level of probability.

**Table 5:** Impact of sewage sludge use with fertilizers on nitrogen concentration and protein content.

Treatments	Nitrogen Concentration (%)			
	2017–2018		2018–2019	
	III-Rice	III-Wheat	IV-Rice	IV-Wheat
T_0_ (WF)	1.28 ± 0.084 c	1.69 ± 0.09 f	1.26 ± 0.07 c	1.33 ± 0.05 e
T_1_ (RDF 100)	1.54 ± 0.11 b	1.90 ± 0.07 cde	1.50 ± 0.10 b	1.95 ± 0.00 bc
T_2_ (RDF 100 + SSL 20)	1.65 ± 0.08 a	2.44 ± 0.07 ab	1.58 ± 0.09 ab	2.15 ± 0.22 ab
T_3_ (RDF 100 + SSL 30)	1.78 ± 0.06 a	2.69 ± 0.7 a	1.66 ± 0.08 a	2.26 ± 0.03 a
T_4_ (RDF 50 + SSL 20)	1.43 ± 0.07 bc	1.72 ± 0.03 ef	1.39 ± 0.04 bc	1.66 ± 0.03 d
T_5_ (RDF 60 + SSL 20)	1.51 ± 0.02 b	1.79 ± 0.03 bc	1.40 ± 0.07 bc	1.72 ± c0.03 d
T_6_ (RDF 70 + SSL 20)	1.55 ± 0.52 b	1.88 ± 0.04 cde	1.47 ± 0.05 bc	1.80 ± 0.04 cd
T_7_ (RDF 50 + SSL 30)	1.55 ± 0.00 b	1.89 ± 0.03 cde	1.48 ± 0.04 bc	1.82 ± 0.04 cd
T_8_ (RDF 60 + SSL 30)	1.56 ± 0.08 b	1.96 ± 0.02 cd	1.51 ± 0.059 b	1.85 ± 0.02 cd
T_9_ (RDF 70 + SSL 30)	1.59 ± 0.02 b	2.04 ± 0.00 c	1.54 ± 0.02 b	1.95 ± 0.07 bc
Significance level	**	**	**	**
Protein Content (%)				
T_0_ (WF)	8.03 ± 0.51 c	10.59 ± 0.58 f	7.90 ± 0.48 c	8.31 ± 0.35 e
T_1_ (RDF 100)	9.63 ± 0.71 b	11.85 ± 0.44 cde	9.37 ± 0.66 ab	10.23 ± 0.02 d
T_2_ (RDF 100 + SSL 20)	10.29 ± 0.50 ab	15.23 ± 0.46 b	9.86 ± 0.57 ab	13.42 ± 1.39 ab
T_3_ (RDF 100 + SSL 30)	11.15 ± 0.41 a	16.81 ± 0.46 a	10.35 ± 0.51 a	14.13 ± 0.23 a
T_4_ (RDF 50 + SSL 20)	8.94 ± 0.43 bc	10.75 ± 0.22 ef	8.71 ± 0.30 bc	10.35 ± 0.19 d
T_5_ (RDF 60 + SSL 20)	9.42 ± 0.16 b	11.19 ± 0.20 def	9.03 ± 0.44 abc	10.78 ± 0.23 cd
T_6_ (RDF 70 + SSL 20)	9.71 ± 0.32 b	11.75 ± 0.27 cde	9.17 ± 0.35 abc	11.26 ± 0.27 cd
T_7_ (RDF 50 + SSL 30)	9.69 ± 0.32 b	11.79 ± 0.20 cde	9.25 ± 0.25 abc	11.39 ± 0.25 cd
T_8_ (RDF 60 + SSL 30)	9.73 ± 0.49 b	12.25 ± 0.11 cd	9.46 ± 0.37 ab	11.59 ± 0.17 cd
T_9_ (RDF 70 + SSL 30)	10.04 ± 0.18 b	12.75 ± 0.04 c	9.63 ± 0.12 ab	12.17 ± 0.48 bc
Significance level	**	**	**	**

Mean values within the same column having the same letter differ non-significantly (*p* ≤ 0.01), while different letters indicate a significant difference (*p* ≤ 0.01). Mean (±SE) was taken from three replicates for each treatment. **, indicates significant at 1% level of probability.

**Table 6:** Impact of sewage sludge use with fertilizers on cadmium and chromium concentration in rice and wheat.

Treatments	Grain			
	Cadmium (mg kg^−1^)			
	2017–2018		2018–2019	
	III-Rice	III-Wheat	IV-Rice	IV-Wheat
T_0_ (WF)	0.29 ± 0.04 c	0.33 ± 0.03 d	0.25 ± 0.04 e	0.29 ± 0.05 d
T_1_ (RDF 100)	0.45 ± 0.06 c	0.51 ± 0.03 c	0.42 ± 0.05 d	0.49 ± 0.02 c
T_2_ (RDF 100 + SSL 20)	1.12 ± 0.07 a	1.24 ± 0.03 a	1.10 ± 0.06 ab	1.20 ± 0.05 a
T_3_ (RDF 100 + SSL 30)	1.27 ± 0.12 a	1.36 ± 0.04 a	1.23 ± 0.05 a	1.31 ± 0.01 a
T_4_ (RDF 50 + SSL 20)	0.81 ± 0.07 b	0.89 ± 0.06 b	0.80 ± 0.02 c	0.85 ± 0.05 b
T_5_ (RDF 60 + SSL 20)	0.85 ± 0.09 b	0.94 ± 0.03 b	0.83 ± 0.05 c	0.91 ± 0.06 b
T_6_ (RDF 70 + SSL 20)	0.87 ± 0.07 b	0.95 ± 0.04 b	0.83 ± 0.02 c	0.92 ± 0.05 b
T_7_ (RDF 50 + SSL 30)	1.10 ± 0.06 a	1.24 ± 0.07 a	1.05 ± 0.06 ab	1.18 ± 0.04 a
T_8_ (RDF 60 + SSL 30)	1.14 ± 0.05 a	1.25 ± 0.06 a	1.11 ± 0.02 ab	1.21 ± 0.06 a
T_9_ (RDF 70 + SSL 30)	1.19 ± 0.04 a	1.26 ± 0.08 a	1.16 ± 0.04 ab	1.23 ± 0.01 a
Significance level	**	**	**	**
	Chromium (mg kg^−1^)			
T_0_ (WF)	1.37 ± 0.033 b	1.21 ± 0.25 c	1.17 ± 0.092 e	1.04 ± 0.10 g
T_1_ (RDF 100)	1.97 ± 0.18 b	1.75 ± 0.09 c	1.46 ± 0.36 e	1.27 ± 0.14 g
T_2_ (RDF 100 + SSL 20)	4.78 ± 0.67 a	4.22 ± 0.17 a	3.55 ± 0.058 b	3.24 ± 0.14 bc
T_3_ (RDF 100 + SSL 30)	4.90 ± 0.67 a	4.49 ± 0.36 a	4.11 ± 0.51 a	3.55 ± 0.03 a
T_4_ (RDF 50 + SSL 20)	4.25 ± 0.79 a	3.54 ± 0.34 b	2.92 ± 0.14 d	2.50 ± 0.08 f
T_5_ (RDF 60 + SSL 20)	4.41 ± 0.59 a	3.68 ± 0.26 ab	3.01 ± 0.06 d	2.67 ± 0.10 ef
T_6_ (RDF 70 + SSL 20)	4.49 ± 0.29 a	3.79 ± 0.22 ab	3.04 ± 0.03 cd	2.68 ± 0.01 ef
T_7_ (RDF 50 + SSL 30)	4.64 ± 0.63 a	3.91 ± 0.16 ab	3.29 ± 0.10 bcd	2.90 ± 0.06 de
T_8_ (RDF 60 + SSL 30)	4.79 ± 0.82 a	4.05 ± 0.18 ab	3.50 ± 0.11 bc	3.02 ± 0.08 cd
T_9_ (RDF 70 + SSL 30)	4.85 ± 0.69 a	4.34 ± 0.43 ab	4.04 ± 0.10 a	3.47 ± 0.11 ab
Significance level	**	**	**	**

Mean values within the same column having the same letter differ non-significantly (*p* ≤ 0.01), while different letters indicate a significant difference (*p* ≤ 0.01). Mean (±SE) was taken from 3 replicates for each treatment. **, indicates significant at 1% level of probability.

**Table 7:** Impact of sewage sludge use with fertilizers on nickel and lead concentration in rice and wheat.

Treatments	Grain			
	Nickel (mg kg^−1^)			
	2017–2018		2018–2019	
	III-Rice	III-Wheat	IV-Rice	IV-Wheat
T_0_ (WF)	7.65 ± 0.67 c	7.93 ± 0.36 d	7.35 ± 0.52 c	6.28 ± 0.87 c
T_1_ (RDF 100)	9.70 ± 0.95 c	9.12 ± 0.48 d	9.05 ± 0.83 bc	8.04 ± 1.29 b
T_2_ (RDF 100 + SSL 20)	16.35 ± 0.76 ab	14.62 ± 0.37 bc	14.05 ± 1.10 a	13.32 ± 0.45 a
T_3_ (RDF 100 + SSL 30)	19.00 ± 1.75 a	17.31 ± 1.12 a	15.48 ± 1.36 a	14.35 ± 1.35 a
T_4_ (RDF 50 + SSL 20)	13.96 ± 1.26 b	12.72 ± 0.79 b	12.08 ± 0.96 bc	9.20 ± 1.11 b
T_5_ (RDF 60 + SSL 20)	14.63 ± 0.65 b	12.90 ± 0.38 b	12.12 ± 1.67 bc	9.61 ± 0.36 b
T_6_ (RDF 70 + SSL 20)	14.94 ± 0.76 b	13.68 ± 0.81 bc	12.20 ±1.10 bc	9.26 ± 0.93 b
T_7_ (RDF 50 + SSL 30)	15.06 ± 1.76 b	14.73 ± 0.92 bc	13.23 ± 0.41 a	11.09 ± 0.71 ab
T_8_ (RDF 60 + SSL 30)	16.00 ± 0.79 ab	14.42 ± 0.41 bc	13.47 ± 0.52 a	10.48 ± 0.96 ab
T_9_ (RDF 70 + SSL 30)	17.67 ± 1.46 ab	15.83 ± 1.32 ab	14.58 ± 1.69 a	12.04 ± 1.19 ab
Significance level	**	**	**	**
Treatments	Lead (mg kg^−1^)			
T_0_ (WF)	0.49 ± 0.073 e	0.40 ± 0.08 e	0.40 ± 0.12 c	0.37 ± 0.08 d
T_1_ (RDF 100)	0.85 ± 0.04 d	0.67 ± 0.17 d	0.59 ± 0.03 c	0.59 ± 0.03 d
T_2_ (RDF 100 + SSL 20)	1.63 ± 0.087 b	1.51 ± 0.04 b	1.31 ± 0.7 b	1.16 ± 0.08 bc
T_3_ (RDF 100 + SSL 30)	1.99 ± 0.15 a	1.93 ± 0.04 a	1.81 ± 0.10 a	1.53 ± 0.25 a
T_4_ (RDF 50 + SSL 20)	1.30 ± 0.10 c	1.23 ± 0.08 c	1.13 ± 0.05 b	0.94 ± 0.03 c
T_5_ (RDF 60 + SSL 20)	1.32 ± 0.03 c	1.28 ± 0.06 bc	1.13 ± 0.03 b	0.99 ± 0.04 c
T_6_ (RDF 70 + SSL 20)	1.41 ± 0.04 bc	1.37 ± 0.07 bc	1.19 ± 0.05 b	0.99 ± 0.03 c
T_7_ (RDF 50 + SSL 30)	1.51 ± 0.04 bc	1.40 ± 0.04 bc	1.26 ± 0.10 b	1.15 b ± 0.02 c
T_8_ (RDF 60 + SSL 30)	1.53 ± 0.14 bc	1.44 ± 0.06 bc	1.27 ± 0.06 b	1.16 ± 0.07 bc
T_9_ (RDF 70 + SSL 30)	1.92 ± 0.07 a	1.86 ± 0.07 a	1.69 ± 0.01 a	1.39 ± 0.13 ab
Significance level	**	**	**	**

Mean values within the same column with the same letter differ non-significantly (*p* ≤ 0.01), while different letters indicate a significant difference (*p* ≤ 0.01). Mean (±SE) was taken from 3 replicates for each treatment. **, indicates significant at 1% level of probability.

## Data Availability

Data are available after request.
